# Single-session laparoscopic cystectomy and nephroureterectomy: is it real and useful choice of treatment or fiction?

**DOI:** 10.1093/jscr/rjab409

**Published:** 2021-09-09

**Authors:** Raffaele Baio, Giovanni Molisso, Alessandro Pane, Umberto Di Mauro, Oliviero Intilla, Roberto Sanseverino

**Affiliations:** Department of Medicine and Surgery ‘Scuola Medica Salernitana’, University of Salerno, Baronissi, Salerno, Italy; Department of Urology, Umberto I Hospital, Nocera Inferiore, Salerno, Italy; Department of Urology, Umberto I Hospital, Nocera Inferiore, Salerno, Italy; Department of Urology, Umberto I Hospital, Nocera Inferiore, Salerno, Italy; Department of Urology, Umberto I Hospital, Nocera Inferiore, Salerno, Italy; Department of Urology, Umberto I Hospital, Nocera Inferiore, Salerno, Italy

## Abstract

Patients with recurrent high grade and/or muscle-invasive bladder cancer and concomitant upper urinary tract disease, e.g. urothelial tumors or afunctional hydronephrotic kidneys, may be candidates for simultaneous laparoscopic cystectomy and nephroureterectomy. So, such patients, especially when affected by multiple comorbidities, can benefit from the avoidance of extended laparotomy. We report our experience with simultaneous laparoscopic radical cystectomy and right nephroureterectomy in a 67-year-old-male patient affected by recurrent polyfocal high grade bladder cancer and an associated right upper tract carcinoma. This laparoscopic approach was technically successful without the need for conversion to open surgery. More than a year after the surgery, the patient is still alive, showing no tumor relapse of at the established instrumental controls. This laparoscopic approach, performed in a single session, can be safe and feasible in selected cases as an alternative approach to the open surgery, offering good oncological and functional results.

## INTRODUCTION

Urothelial cancer is the fourth most common malignancy in developed countries, affecting more frequently the urinary bladder and in only 5–10% of cases the upper urinary tract [1]. Furthermore, an association between these two cancers was found in the 17% of cases [2]. Open radical cystectomy is still considered the gold standard treatment for muscle-invasive or high risk and recurrent non-muscle-invasive bladder tumors, Bacillus Calmette-Guerin (BCG) refractory, relapsing and unresponsive T1G3 tumors [3, 4]. Open radical nephroureterectomy with bladder cuff excision is the standard treatment for high risk upper urinary tract cancer [5]. Simultaneous nephroureterectomy and radical cystectomy can be performed in patients affected by recurrent high grade or muscle-invasive bladder cancer and concomitant upper urinary tract cancer or non-functional kidney [6]. The first laparoscopic nephroureterectomy was performed in 1991 by Clayman *et al.* [7], whereas the first laparoscopic radical cystectomy dates back to 1992 by Parra *et al.* [8]. Thereafter, probably owing to technical progress, several studies have shown the advantages of laparoscopic approach (compared with open techniques), especially when performed by experienced surgeons. These advantages include: fewer intraoperative and postoperative complications, decreased intraoperative blood loss, less need of analgesics, shorter hospital stay and earlier recovery [9–11], having at the same time functional and oncological results similar to those of open surgery [12, 13]. Several years after, Berglund *et al.* [14] performed laparoscopic radical cystoprostatectomy and bilateral nephroureterectomy, demonstrating that it is a reproducible and oncologically safe technique. We report our experience with simultaneous laparoscopic radical cystectomy and right nephroureterectomy, explaining in Supplementary Video every single step of such a challenging operation.

## CASE REPORT

**Figure 1:**
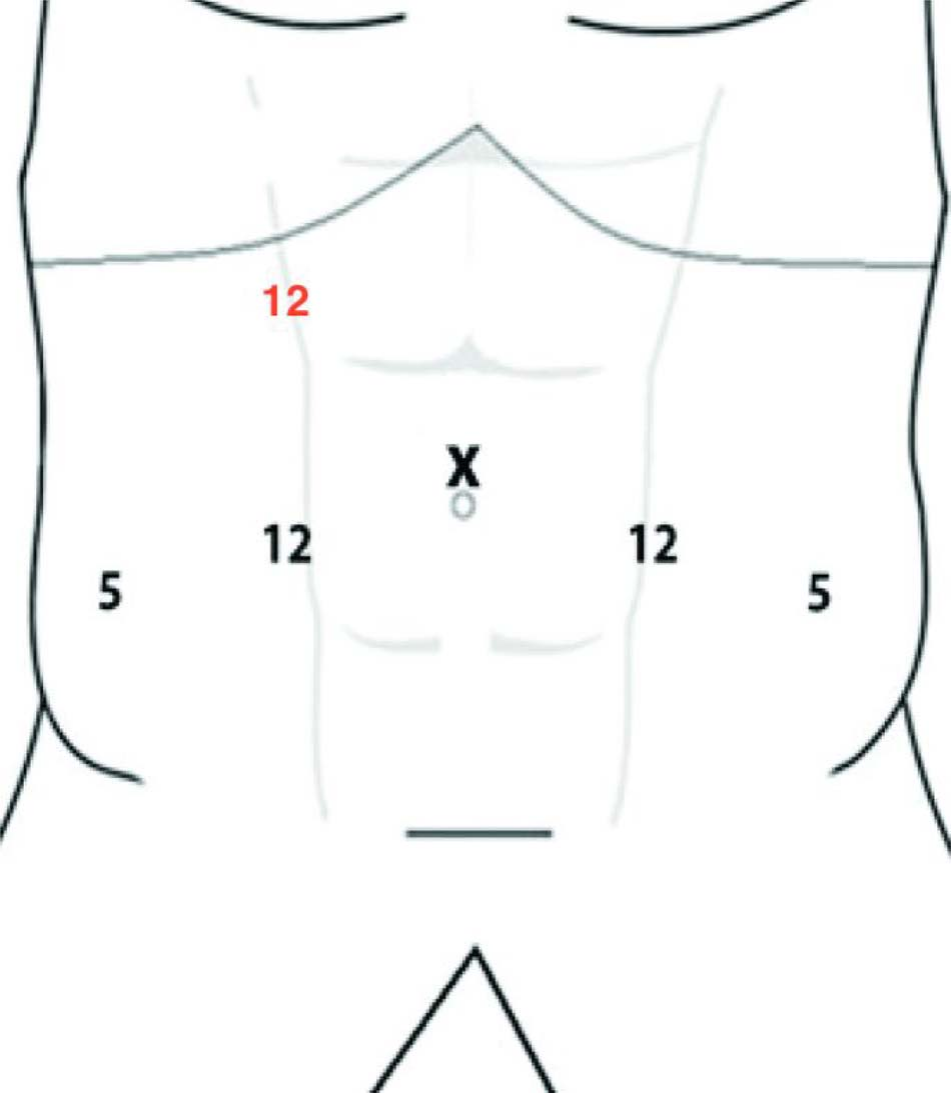
Trocars arrangement.

A 67-year-old male patient affected by recurrent polyfocal high grade bladder cancer and an associated renal pathology (right upper tract carcinoma of 16 mm in diameter on CT scan) underwent simultaneous radical cystectomy and nephroureterectomy with pelvic and lombo-caval lymph node dissection performed by laparoscopic approach in March 2020. According to the pre-operative imaging study, the two tumors were organ-confined. The Clavien–Dindo classification was used to evaluate post-operative complications. We performed laparoscopic transperitoneal approach, using the trocars arrangement shown in the Fig. 1. After positioning a catheter into the bladder, the patient was first placed in right lateral decubitus for the right nephroureterectomy. After inducing pneumoperitoneum using a Verres needle, a 12-mm trocar (used as the camera port and indicated in the figure as ‘X’) was placed 2 cm laterally to the right of the umbilicus. The other two 12-mm trocars were placed in line, in the right pararectal area. During this surgical procedure the renal artery and the renal vein were identified, clamped with Hem-o-lock clips and sectioned between. A perifascial dissection of the kidney was performed, preserving the adrenal gland. Para-caval lymphadenectomy was also performed. For the next step of the surgery, radical cystectomy and bilateral pelvic lymph node dissection, the patient was positioned in dorsal decubitus, in a Trendelenburg position. The camera trocar was the same as for the right nephroureterectomy. Three other trocars (two 5-mm and one 12-m trocars) were placed in addition. The 12-mm trocar was placed, inferior to the umbilicus, in the left pararectal area whereas the two 5-mm trocars were placed in the left and right lower quadrant, proximal to the anterior–superior iliac spine. For bladder dissection, as shown in the Supplementary Video, its vascular peduncles were secured with mechanical stapler and divided. In this way, the lateral plane was dissected, bilaterally. Finally, the urethra was divided distal from the prostatic apex using cold scissors. The pelvic lymphadenectomy was performed around the iliac vessels and obturatory fossa bilaterally. Two tubular drains were used, one in the right renal lodge and the other in the pelvic cavity. Urinary diversion as a unilateral ureterocutaneostomy was constructed by pulling the left ureter through the hand port incision (specifically using the 12-mm trocar on the left side). The ureter was catheterized with a mono J stent. All specimens were placed in an endobag, removed through a midline incision and sent to the pathological examination. The operative field was inspected for bleeding or injury. Peri-operative and post-operative data are shown in Table 1. The pathological stages are represented in Table 2. After discharge, the patient returned to his normal activities without limitations after 3 weeks. More than a year after surgery, the patient is still alive, showing no tumor relapse of at the established instrumental controls.

**Table 1:** Peri-operative and post-operative data

Number of trocars	6
Operative time (min)	360
Blood loss (mL)	850
Hospital stay (days)	13
Surgical margins status	Tumor-free
Follow-up period (months)	16

**Table 2:** Pathological stages

Bladder pathological stage	pT2G3
Number of pelvis lymph nodes dissected	16
Pelvis lymph nodes status	pN0
Upper urinary tract pathological stage	pT1G3
Number of lombo-caval lymph nodes dissected	6
Pelvis lymph nodes status	pN0

## DISCUSSION

We successfully performed laparoscopic radical nephroureterectomy and cystectomy with lombo-caval and pelvic lymph node dissection in a single-session, without the need for conversion to open surgery. According to literature data, minimally invasive surgery can minimize the complications and improve the recovery [9–11]. Performing a similar surgery with a laparoscopic approach is very demanding. To have oncological safety, intraoperatively, particular attention must be paid to avoid tumor leakage. In addition, the sample must be extracted en bloc immediately into an endobag, with the bladder neck closed to avoid contact of the urine with the abdominal cavity. Another important oncological aspect is the extent of lymphadenectomy. According to literature data, extended lymph node dissection during radical cystectomy is possible even when a minimally invasive approach is chosen [15]. We removed 22 lymph nodes. Several studies showed that the oncological safety of a laparoscopic approach is similar to that of open surgery [12, 13]. Although an open surgery including nephroureterectomy and radical cystectomy involve one large midline incision with greater morbidity and longer convalescence, the laparoscopic approach implicates very small trocar incisions and an incision of ~4–5 cm to remove the specimen. In effect, the specimen can be removed through a small lower midline incision, Pfannenstiel incision or transvaginally in female patients. We preferred a small midline incision for the specimen removal due to the lower risk of evisceration. According to small series, performing in a single session laparoscopic nephroureterectomy and cystectomy is feasible, with good oncological results and early recovery [9–11, 12, 13]. A large-scale prospective study will be necessary to provide more information on this surgery in the future. In conclusion, the laparoscopic approach is widely spreading in urology and, in some cases, it has become a standard of care. In selected cases, performing in a single-session laparoscopic radical cystectomy and nephroureterectomy is oncologically safe and technically reproducible, offering oncological and functional results similar to those of open surgery. In addition, choosing a minimally invasive approach, the cosmetic results are better, also with faster post-operative recovery and lower bleeding rates.

## Supplementary Material

New_video_rjab409
